# Cannabinoides en porros de resina de cannabis decomisados en Castilla y León

**DOI:** 10.23938/ASSN.1126

**Published:** 2025-09-26

**Authors:** Eduardo Tejedor-Tejada, Jesús Miguel Tejedor Muñoz

**Affiliations:** 1 Hospital General de Segovia Servicio de Farmacia Hospitalaria Segovia España; 2 Universidad Internacional de La Rioja Logroño La Rioja España; 3 Delegación del Gobierno en Castilla y León Área de Sanidad y Política Social Valladolid España

**Keywords:** Cannabinoides, Dronabinol, Cromatografía de Gases, Fumar Hachís, Policía, Cannabinoids, Dronabinol, Chromatography, Gas, Marijuana smoking, Law Enforcement Officers

## Abstract

**Fundamento:**

El cannabis, en sus productos hierba y resina, es la droga ilegal más consumida en el mundo. El objetivo del estudio es conocer la riqueza y disponibilidad de los principales cannabinoides en la resina de cannabis en los porros circulantes a nivel regional.

**Métodos:**

Se realizó un estudio descriptivo de porros enteros circulantes incautados por las fuerzas de seguridad en la calle en el periodo 2017 a 2020 en la Comunidad Autónoma de Castilla y León. Se determinó el peso bruto (porro completo) y neto (consumible) y mediante cromatografía de gases se analizó la disponibilidad (mg) de los cannabinoides delta-9-Tetrahidrocannabinol (THC), cannabidiol (CBD) y cannabinol (CBN) para obtener la riqueza/potencia (%) de las muestras y estimar su biodisponibilidad (10% y 25%).

**Resultados:**

Se analizaron 639 porros mezcla de resina de cannabis con tabaco (34,3% de los porros enteros incautados). Los pesos bruto y neto mostraron tendencias ligeramente descendentes (6,5-8,5%). Las medianas de riqueza para THC, CBD y CBN fueron 7,58%, 0,62% y 0,60%, y las de disponibilidad 57,04 mg, 4,57 mg y 4,46 mg. Tanto la riqueza como la disponibilidad presentaron una tendencia menguante a lo largo del periodo de estudio para THC (13,5%), CBN (73%) y CBD (52-67%).

**Conclusión:**

La riqueza y disponibilidad de THC en porros incautados de resina de cannabis en el periodo de estudio, superiores a los descritos en porros de cannabis, alertan de una alta exposición del consumidor y de posibles consecuencias sanitarias a pesar de la tendencia decreciente de la riqueza.

## Abstract (en)

**Background:**

Cannabis, in both its herbal form and resin-derived products, is the most widely consumed illicit drug worldwide. This study aimed to determine the potency and dosage of the primary cannabinoids present in cannabis resin from joints circulating in the region.

**Methods:**

A descriptive study was conducted on seized joints confiscated by law enforcement agencies in the Autonomous Community of Castilla y León (Spain) between 2017 and 2020. Gross (whole joint) and net (filler) weights were recorded. Gas chromatography was used to quantify cannabinoids delta-9-tetrahydrocannabinol, cannabidiol, and cannabinol. Potency (percentage content) and dosage (mg) were calculated and bioavailability was estimated at 10% and 25%.

**Results:**

A total of 639 joints containing a mixture of cannabis resin and tobacco were analyzed (34.3% of confiscated samples). Both gross and net weights showed a decreasing trend over time (6.5-8.5%). Median potency values for delta-9-tetrahydrocannabinol, cannabidiol, and cannabinol were 7.58%, 0.62%, and 0.60%, respectively, while corresponding dosages were 57.04 mg, 4.57 mg, and 4.46 mg. Overall potency and dosage declined across the study period for THC (13.5%), CBN (73%) and CBD (52-67%).

**Conclusions:**

The delta-9-tetrahydrocannabinol potency and dosage found in confiscated cannabis resin joints during the study period were higher than those typically reported for herbal cannabis joints, suggesting greater user exposure and increased potential health risks.

## INTRODUCCIÓN

El cannabis sigue siendo la sustancia psicoactiva más consumida en todo el mundo. En el año 2022, había 228 millones de consumidores de cannabis entre 15 y 64 años, lo que corresponde a más del 4,4% de la población mundial^1^.

En España es la droga con mayor prevalencia de consumo en la población de 15 a 64 años. Históricamente, el consumo de cannabis hierba o marihuana está más extendido (53,8%) que el de cannabis resina o hachís (21,7%); este último está estrechamente relacionado con el consumo de tabaco: el 90,6% de las personas consumidoras en los últimos 30 días reconoce haberlo consumido mezclándolo con tabaco^2^.

El consumo de drogas está reconocido como un factor contribuyente a la carga de morbimortalidad global. La alta prevalencia del consumo de cannabis (3,7 millones de consumidores europeos diarios) hace que los problemas de salud relacionados con el mismo puedan tener implicaciones para la salud pública^3^.

El consumo de cannabis se describe primordialmente por la frecuencia de uso, aunque la dosis es fundamental para evaluar los efectos vinculados con las drogas^4^^,^^5^. La frecuencia y la cantidad de consumo se asocian positivamente con problemas relacionados con el cannabis y se considera que la cantidad, más que la frecuencia, es un predictor importante de dichos problemas^5^^,^^6^.

La mayoría de estudios se han realizado desde la perspectiva de la frecuencia de uso. Además, el número de trabajos enfocados en la cantidad de consumo es reducido, lo que obstaculiza una mejor comprensión del daño relacionado con el cannabis. Las limitaciones de las investigaciones sobre el porcentaje y la cuantificación (en miligramos, mg), de los cannabinoides principales de los porros son fundamentalmente debidos a problemas de accesibilidad a las muestras y, por otra parte, a que los instrumentos de autoinforme son inadecuados o tienen una validez relativa^4^^,^^7^.

El objetivo de esta investigación es conocer la potencia (%) y la dosis en mg de Δ-9-tetrahidrocannabinol (THC), cannabinol (CBN) y cannabidiol (CBD) de los porros de resina de cannabis circulantes en la Comunidad Autónoma de Castilla y León. Este conocimiento es clave para estimar el grado de exposición del consumidor, y aportar información relevante al diseño de las políticas de drogas y salud pública.

## MATERIAL Y MÉTODOS

Se realizó un estudio observacional transversal de los cigarros enteros circulantes tipo porro de resina de cannabis decomisados a nivel de la calle por las Fuerzas de Seguridad durante el periodo 2017 a 2020, procedentes de las nueve provincias de la Comunidad Autónoma de Castilla y León.

Se incluyeron exclusivamente los porros enteros, sin manipulación adicional alguna, que en su composición contuvieran de forma aparente resina de cannabis, excluyéndose los porros enteros con cannabis hierba o marihuana.

Todas las muestras requisadas que cumplían con los criterios de selección se enviaron al laboratorio del Área de Sanidad de la Delegación del Gobierno en la Comunidad Autónoma de Castilla y León, laboratorio oficial en nuestro ámbito territorial para el análisis de drogas, tanto en procesos judiciales como en procesos administrativos sancionadores.

Las variables investigadas fueron el peso bruto del porro entero (totalidad del porro con su continente y contenido), peso neto/consumible (muestra sin boquilla/filtro ni papel), determinación de la riqueza y dosis en mg de THC, CBN y CBD en cada porro, y la biodisponibilidad de THC según dos escenarios, de 10% y 25%, por considerarse los más realistas según la bibliografía consultada^8^^-^^11^.

Con fines comparativos, también se determinaron la potencia/riqueza y dosis de THC en las pastillas de resina sin manipular incautadas durante el periodo de estudio.

### Análisis cualitativo y cuantitativo de laboratorio

Para determinar el peso bruto se procedió al pesaje del porro completo incluido papel y boquilla o filtro. Como peso neto/consumible/aprovechable se tomó el peso de la sustancia (producto marrón), con o sin tabaco, que constituye el porro. En ambas operaciones se utilizó una balanza (Mettler Toledo AE200S) con precisión de 0,1mg.

Para la determinación presuntiva de cannabinoides (cualitativa) se siguió el manual publicado para el cannabis^12^: Se coloca el material (producto marrón) con tabaco, en un tubo de ensayo, al que se le añaden 2 mL de reactivo Duchenois-Levine y, a continuación, 0,5 mL de HCl 37%; por último, se añade 1,0 mL de cloroformo. Se forman dos fases, quedando la fase de cloroformo en el fondo del tubo. Se consideran positivas las muestras que presentan coloración azul en la fase clorofórmica.

Para la determinación cuantitativa de cannabinoides se pesaron 10 mg de la muestra, previa homogenización. Para minimizar los errores asociados al error de la balanza, se utilizó el método de doble pesada por el que se toman dos porciones de la sustancia y se pesan independientemente. Cada una de las porciones pesadas se llevó a un matraz de 10 mL (precisión 0,025 mL) y se enrasó con etanol absoluto(99,5%). Se homogenizaron mediante agitación magnética durante veinte minutos y se filtraron con un filtro de jeringa con membrana de polivinilideno (PVDF) con diámetro de 25 mm y tamaño del poro de 0,45 μm, para eliminar impurezas y proteger la columna del equipo. La determinación de la pureza de THC se llevó a cabo en un cromatógrafo de gases con detector de ionización de llama (modelo HP 7890A) e inyector automático (modelo G4513A CN 10430050), con interfaz del equipo *Chemstation* versión B.04.03 y cuyas condiciones cromatográficas fueron:

- Volumen de inyección (automático) 1μl, portal de inyección 4,2 psi, flujo 289,38 mL/min, gas portador helio;
- Columna de polisiloxano semicapilar HP-1, de 5 m de largo, 0,535 mm de diámetro interno y 2,65 µm de espesor de película;
- Temperatura del portal de inyección: 290 ºC, temperatura del horno: 200 ºC 1,5 minutos, rampa 40 ºC/min hasta alcanzar 270 ºC de temperatura final en el detector.

La inyección en el equipo del método se realizó en modo Split 10:1^12^. En estas condiciones, los tiempos de retención de las distintas moléculas son 2.858 min para THC; 2.628 min para CBD y 2.998 min para CBN, obteniéndose cromatogramas con picos bien definidos que permiten confirmar la presencia de los correspondientes cannabinoides.

La recta de calibrado se realizó a partir de diluciones de patrones certificados entre 1,0 ppm y 55 ppm para THC, CBN y CBD. El coeficiente de correlación de Pearson fue de 0,9998 para THC, 0,9978 para CBN y 0,9973 para CBD. El límite de detección es 0,36 ppm y de cuantificación 1,09 ppm. La introducción de patrones de concentración conocida en la secuencia de trabajo permite validar las condiciones cromatográficas.

La altura/área del pico determina la riqueza o % del cannabinoide (THC, CBN, CBD) en la muestra, que se calcula como la media de los resultados obtenidos para cada una de las porciones inyectadas de la misma muestra. Se fija, como especificación, que la diferencia entre los valores obtenidos de las dos porciones no supere el 5%.

La riqueza (%) de cannabinoide permite calcular su disponibilidad (cantidad en mg de la molécula) a partir del peso de la muestra en mg:

**mg de cannabinoide en la muestra = peso neto (mg) ▪ % de cannabinoide**

Se elaboró una base de datos de las concentraciones obtenidas de THC, CBD y CBN en todas las muestras judiciales de porros de resina de cannabis y de THC en todas las muestras de resina de cannabis incautadas entre los años 2017 y 2020 en la Comunidad Autónoma de Castilla y León.

### Análisis estadístico

Las variables categóricas se describieron como frecuencias y porcentajes. Las variables continuas mediante la media y la desviación estándar, y la mediana y el rango intercuartílico (RIC), según sigan o no una distribución normal, comprobada mediante la prueba de Kolmogorov-Smirnov. Los valores atípicos fueron identificados a través del supuesto de Tukey^13^. El análisis estadístico descriptivo se realizó mediante el programa IBM Statistics (SPSS.v.25).

## RESULTADOS

**Figura 1:**
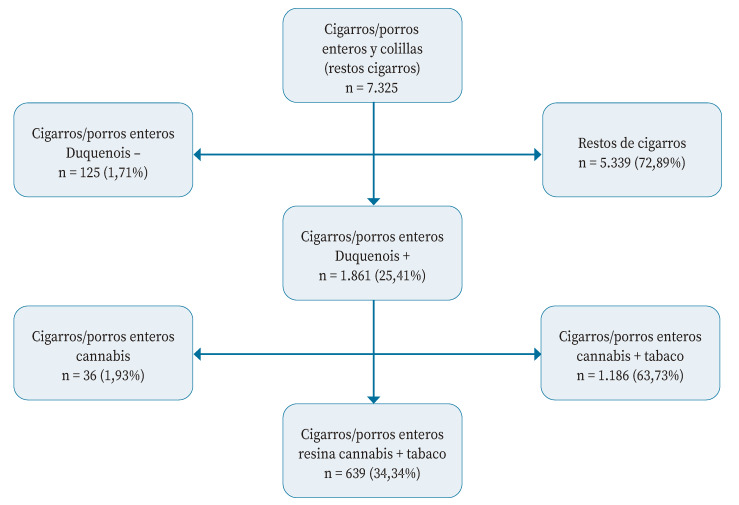
Proceso de selección de las 639 muestras incautadas analizadas.

Se decomisaron 7.325 cigarros enteros y restos de cigarros en la comunidad autónoma de Castilla y León durante el periodo de estudio; no se incautaron pipas, ni productos para consumo oral o vaporizadores. Una vez excluidas las muestras no relacionadas con el objetivo del estudio, se analizaron 1.861 porros enteros, una tercera parte de los cuales eran mezcla de resina de cannabis con tabaco (Figure 1).

Los 639 porros de resina de cannabis estudiados contenían nicotina; el 99,84% contenían THC, el 96,24% CBN y el 85,13%CBD.

Los resultados analíticos globales de la comunidad autónoma de Castilla y León en los años examinados de los porros de resina de cannabis mezclada con tabaco se muestran en la Tabla 1.

**Tabla 1:** Características de los porros de resina (n=639) decomisados entre 2017 y 2020

Peso (g)	Media (DE)	Rango	Mediana (RIC)	CV (%)
Bruto	0,96 (0,28)	2,39	0,93 (0,31)	29,2
Neto/consumible	0,77 (0,25)	2,32	0,73 (0,24)	32,5
THC				
Peso (mg)	65,15 (44,88)	340,56	57,04 (46,92)	68,9
%	8,39 (4,70)	28,17	7,58 (6,48)	56
Biodisponibilidad (mg)				
10%	6,52 (4,49)	34,06	5,70 (4,69)	68,9
25%	16,29 (11,22)	85,14	14,26 (11,73)	68,9
CBN				
Peso (mg)	6,12 (6,08)	56,83	4,57 (6,01)	99,3
%	0,79 (0,66)	4,06	0,62 (0,73)	83,5
CBD				
Peso (mg)	5,80 (5,82)	48,59	4,46 (6,27)	100
%	0,75 (0,74)	6,17	0,60 (0,80)	98,7

DE: desviación estándar; RIC: rango intercuartilico; CV: coeficiente de variación; THC: tetrahidrocannabinol; CBD: canabidiol; CBN: cannabinol; %: riqueza/potencia del porro en ese cannabinoide; mg: disponibilidad del cannabinoide.

**Figura 2:**
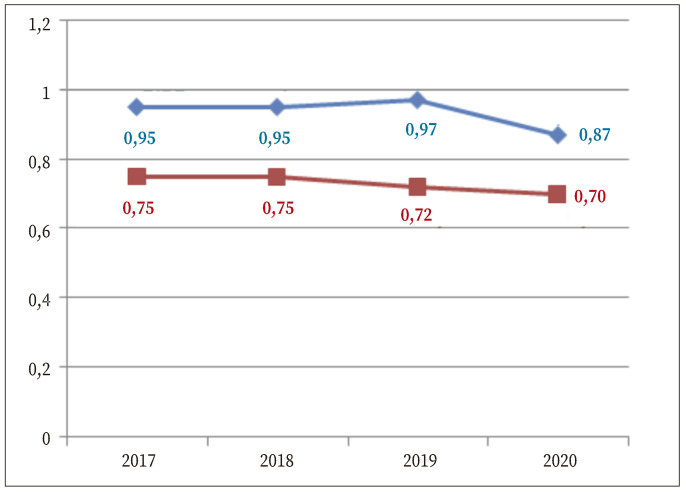
Evolución del peso (g) total (azul) y consumible (rojo) de los porros mezcla de resina y tabaco incautados en el periodo 2017-2020.

El peso total/bruto y el peso neto de los porros de resina decomisados en nuestra comunidad autónoma durante los años investigados presentaron una tendencia descendente (Figura 2). Mientras que el peso neto mediano apenas disminuyó un 6,7%, el peso bruto mediano se mantuvo alrededor de 0,95 hasta 2020, año en el que disminuyó un 8,4%.

**Figura 3:**
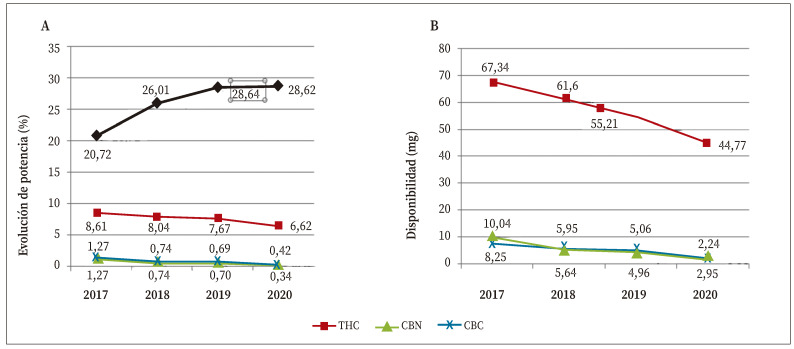
A. Evolución de la potencia (%) de THC en decomisos de resina de cannabis sin manipular (línea negra) y de los principales cannabinoides (THC, CBN, CBD) en porros de resina durante el periodo 2017-2020. B. Evolución de la disponibilidad (mg) de cannabinoides en porros de resina durante el mismo periodo.

La potencia (%) de THC y su disponibilidad (mg) en porros decrecieron desde 2017 a 2020, especialmente en el último año: 13,69% la potencia y 13,48% la disponibilidad (mg). También la riqueza y la disponibilidad de CBN disminuyeron de forma progresiva un 73,22% y un 72,8%, respectivamente, a lo largo de los cuatro años, de forma similar y más acusada que la riqueza y disponibilidad de CBD (66,9% y 52,4%) (Figura 3).

Durante los 4 años estudiados se incautaron 978 piezas de resina sin manipular. La riqueza (%) de THC mostró un incremento del 38,2% en los dos primeros años, estabilizándose en los dos últimos (Figura 3A). Sin embargo, la disponibilidad (mg) disminuyó de forma progresiva un 29,1% a lo largo de los cuatro años (Figura 3B).

## DISCUSIÓN

La investigación realizada sobre 639 muestras tipo porro supera la casuística de otros estudios similares, 232 porros de cannabis con tabaco y 83 unidades porro de resina de cannabis con tabaco^14^ y 98 porros de marihuana con tabaco^15^. En la búsqueda bibliográfica efectuada no se ha encontrado estudios con porros enteros incautados por las fuerzas de seguridad.

El estudio muestra que los valores del THC en los porros de resina cannabis tienen una distribución no paramétrica, similar a la reflejada en otra publicación^14^, y se utilizó la mediana como dato estadístico más adecuado.

La encuesta sobre Alcohol y Drogas en España 2024^2^ recoge que el 98,8% de los consumidores de cannabis de edad comprendida entre 15 y 64 años manifestaron su preferencia de consumo por el porro o canuto y en mucha menor proporción usan pipas de agua (3,9%), vía oral (1,3%) o *vapers* (0,4%).

Los porros incautados de resina de cannabis/hachís en Castilla y León utilizan una ruta de administración mayoritaria de mezcla con tabaco que representa el 100 % de las muestras confiscadas en este estudio, ello ratifica la afirmación *la práctica cotidiana del consumo de cannabis en España es que no existe porro sin tabaco*^16^, aunque si la referencia es la totalidad de los porros enteros confiscados supone que el 98,07% están mezclados con tabaco. En Europa, la vía más utilizada para consumir productos de cannabis(marihuana y hachís)también es la combinación con tabaco, aunque con un porcentaje inferior, con valores comprendidos entre el 77,2% del Reino Unido y el 90,9% de Suiza. Sin embargo en países de América y Oceanía emplean una vía de administración de consumo sin tabaco que oscila entre 92,1% de Estados Unidos, el 79,2% de Canadá y del 70,2% en Nueva Zelanda^17^. Esta práctica de consumo simultaneo aumenta los riesgos derivados del consumo de ambas sustancias^18^ y expone a los consumidores a dos sustancias psicoactivas, la nicotina y el tetrahidrocannabinol, por lo que se puede considerar como policonsumo.

La mediana del peso de los porros mezclados (hachís con tabaco) fue similar a la de otro estudio (0,90 g)^19^, pero existen resultados dispares, superiores (0,94 g; rango: 0,56-1,76)^15^ e inferiores (0,80 g)^20^.

El peso neto del porro (excluida la boquilla/filtro y papel de fumar), constituye el elemento esencial para calcular la dosis en mg de THC del porro de forma sistematizada y objetiva, pues la marihuana/hachís mezclado con tabaco en un porro es muy variable, dependiendo del tipo de consumidor. También es variada la forma de dar el peso neto de la marihuana en el porro: 0,32 g^21^; rangos de 0,3 a 0,5 g^22^; 0,46 g (IC95%: 0,43-0,50)^23^; 0,26 g (RIC=0,13)^14^; y 0,26 g (rango: 0,07-0,89)^15^; solo una publicación establece la cantidad de hachís por porro (0,25 g)^15^.

La mediana del porcentaje de THC en los porros de resina de cannabis para el periodo investigado fue 7,58% (RIC=6,48). No se ha encontrado trabajos que faciliten la riqueza del THC en porros de resina de cannabis para su comparación. En el periodo analizado se observa un descenso progresivo en los años 2017-2019 que se acentúa en el último año 2020 (13,69%). Esta disminución durante el año 2020 pudiera tener relación con la pandemia de COVID 19 (prevalencia de consumo antes de la pandemia 7,8% y durante la pandemia 6,5%, lo que supone un descenso del 17%, incluido el consumo problemático)^24^.

La dosis de mg de THC del porro es el parámetro fundamental para la valoración de la cantidad consumida y del futuro daño potencial. Los valores obtenidos en nuestra investigación son superiores a los obtenidos en porros de cannabis^14^^,^^15^ y, especialmente, superan muy ampliamente a los 7,94 mg (RIC=10,61) publicados en porros de hachis^14^, lo que podría explicarse no solo por la diferente metodología, sino por tratarse de una resina de cannabis con baja concentración de THC.

A los resultados alcanzados hay que aplicarles la biodisponibilidad del cannabis fumado. Los valores de biodisponibilidad varían entre el 23-27% en fumadores habituales, y el 10-14% en fumadores ocasionales^8^^,^^9^^,^^11^^,^^25^. Solo el 10-25% del THC inhalado pasa al torrente sanguíneo; con un cigarro se absorberían entre 0,2 y 4,4 mg, y la dosis mínima para producir efectos farmacológicos estaría entre 2 y 22 mg^10^^,^^26^. En la hipótesis del trabajo se plantean dos escenarios de biodisponibilidad del cannabis: 10% y 25%. De acuerdo con los estudios descritos, la mediana de biodisponibilidad de los porros mixtos de hachís y tabaco fluctuaría entre 5,70 mg (RIC=4,69) y 14,26 mg (RIC=11,73). No se han encontrado trabajos similares para contrastar los resultados hallados. El Tribunal Supremo de España, tomando como referencia técnica un informe del Instituto Nacional de Toxicologia^27^, ha establecido en 10 mg la dosis mínima psicoactiva (cantidad mínima que resulta necesaria para generar efectos en las funciones físicas y psíquicas de una persona). Los efectos farmacológicos del cannabis vinculado a la cantidad consumida (mg) han sido descritos por investigadores en diferentes estudios^28^^-^^30^. La evidencia epidemiológica también muestra que el consumo de cannabis está asociado con un mayor riesgo de resultados psicóticos y confirma una relación dosis-respuesta entre el nivel de consumo y el riesgo de psicosis posterior^5^^,^^31^^-^^33^.

El papel del CBD como modulador y su interacción con el THC está en investigación. El contenido de CBD **e**n los porros referidos es superior a los descritos en literatura^6^ e inferior a las dosis utilizadas en estudios de investigación clínica^34^^-^^37^.

Este estudio presenta limitaciones como centrarse exclusivamente en el análisis de porros decomisados en la vía o establecimientos públicos. Por tanto, 1) no se aborda el consumo privado en domicilio o clubes cannábicos, 2) puede existir un posible sesgo de las incautaciones policiales debido a la selección de presuntos consumidores en áreas públicas, y 3) el perfil epidemiológico es muy limitado debido a la falta de datos en las denuncias sobre consumidores, al no ser un objetivo policial; se desconoce si el consumidor es esporádico o habitual. Las muestras proceden de las provincias de la Comunidad Autónoma de Castilla y León y se prepararon en un contexto específico; por tanto, no es posible generalizar a otras poblaciones y disminuye su validez externa. El traslado de las muestras desde las diferentes provincias al laboratorio en Valladolid conlleva una demora temporal y la consiguiente degradación del THC.

En conclusión, este estudio alerta de las altas dosis de THC existentes en los porros de resina de cannabis de comisados en Castilla y León, y avisa de los posibles daños que podrían producirse por los usuarios, que ya están sufriendo a tenor de las estadísticas crecientes de urgencias hospitalarias y solicitudes de tratamiento de deshabituación.

Los laboratorios de las Áreas de Sanidad, como centros de análisis de las drogas decomisadas, constituyen una fuente de datos complementaria. Estas unidades aportan y proporcionan datos de sustancias confiscadas en la calle por las fuerzas de seguridad que pueden contribuir al diseño de las políticas de salud pública en relación con el consumo de drogas. Estas pautas de actuación deberían incluir formación de docentes y alumnado adolescente en la detección y riesgos del consumo, cambios normativos de los derivados del cannabis, jornadas de divulgación y concienciación, y fondos de investigación.

## Data Availability

Los datos que respaldan los hallazgos de este estudio están disponibles bajo solicitud razonable a los autores correspondiente.
